# The Blocking of Integrin-Mediated Interactions with Maternal Endothelial Cells Reversed the Endothelial Cell Dysfunction Induced by EVs, Derived from Preeclamptic Placentae

**DOI:** 10.3390/ijms232113115

**Published:** 2022-10-28

**Authors:** Yourong Feng, Qi Chen, Sien Yee Lau, Bridget W. Tsai, Katie Groom, Carolyn J. Barrett, Lawrence W. Chamley

**Affiliations:** 1 Department of Obstetrics and Gynaecology, Faculty of Medical and Health Sciences, University of Auckland, Auckland 1023, New Zealand; 2 Liggins Institute, University of Auckland, Auckland 1023, New Zealand; 3 Department of Physiology, Faculty of Medical and Health Sciences, University of Auckland, Auckland 1023, New Zealand; 4 Hub for Extracellular Vesicles Investigations (HEVI), University of Auckland, Auckland 1023, New Zealand

**Keywords:** extracellular vesicle, placenta, preeclampsia, microparticle, integrin

## Abstract

Placental extracellular vesicles (EVs) have increasingly been recognized as a major mediator of feto-maternal communication. However, the cellular and molecular mechanisms of the uptake of placental EVs by recipient cells are still not well-understood. We previously reported that placental EVs target a limited number of organs in vivo. In the current study, we investigated the mechanisms underlying the uptake of placental EVs into target cells. Placental EVs were derived from explant cultures of normal or preeclamptic placentae. The mechanisms underlying the uptake of placental EVs were elucidated, using the phagocytosis or endocytosis inhibitor, trypsin-treatment or integrin-blocking peptides. The endothelial cell activation was studied using the monocyte adhesion assay after the preeclamptic EVs exposure, with and/or without treatment with the integrin blocking peptide, YIGSR. The cellular mechanism of the uptake of the placental EVs was time, concentration and energy-dependent and both the phagocytosis and endocytosis were involved in this process. Additionally, proteins on the surface of the placental EVs, including integrins, were involved in the EV uptake process. Furthermore, inhibiting the uptake of preeclamptic EVs with YIGSR, reduced the endothelial cell activation. The interaction between the placental EVs and the recipient cells is mediated by integrins, and the cellular uptake is mediated by a combination of both phagocytosis and endocytosis.

## 1. Introduction

Placental extracellular vesicles (EVs) are lipid bilayer-enclosed structures extruded primarily from the placental syncytiotrophoblast, which can be detected in the maternal blood at least as early as 6 weeks of gestation [1]. Placental EVs are categorised into large EVs (150 nm to 1 µm) and small EVs (10–100 nm), based on their size [2]. Placental EVs carry functional proteins, regulatory RNAs, DNA and lipids, and while they carry many overlapping cargos, there are differences in the contents of large and small placental EVs [3,4,5].

Placental EVs have increasingly been recognized as a major mediator of feto-maternal communication, mediating several physiological processes, including maternal cardiovascular adaptations and immunological tolerance to the semi-allogeneic fetus [6,7]. One mechanism impacting the functions of the recipient cells is the uptake of the placental EVs, which then release their cargos into the recipient cells. The cellular uptake of EVs can be mediated by phagocytosis [8] or endocytosis [9,10,11], or by the fusion of EVs with the plasma membrane [12]. However, the EV uptake mechanisms may be recipient cell-specific and EV source-dependent. The details of the cellular and molecular mechanisms of the uptake of placental EVs by recipient cells are not yet fully defined.

Recently, many studies have reported that protein interactions are key in the EV uptake, as this facilitates the subsequent endocytosis [13]. It has recently been reported that the specificity of the recipient cell-EV interactions is likely to be determined by cellular “addressins”, on the surface of the EVs, such as integrins [14,15]. For example, it has recently been shown that placental EVs are targeted to the lungs via Arg-Gly-Asp (RGD)-containing integrins, while other placental EVs, decorated with HYD-1 containing integrins, are targeted to the liver [16].

Integrins are heterodimers that bind to a variety of ligands [17]. Integrins have been studied extensively and are now recognised mediators of the interactions of EVs to target cells [15,17]. Our previous in vivo studies have shown that placental EVs are targeted to specific maternal organs, including the lungs, liver and kidneys and that the vesicles have functional effects, including on the vasculature [11,18]. A proteomic study reported that a large range of integrins (integrin α1, αIIb, α5, α6, αM, β1, β2, β3 and β5) were present in placental EVs [4]. Therefore, to better understand the mechanisms underlying the uptake of placental EVs, we undertook this study to investigate whether integrins are involved in the uptake of placental EVs, and whether this mechanism is recipient cell specific.

## 2. Results

### 2.1. Characterization of Large and Small Extracellular Vesicles Derived from Human Term Placenta

**Figure 1:**
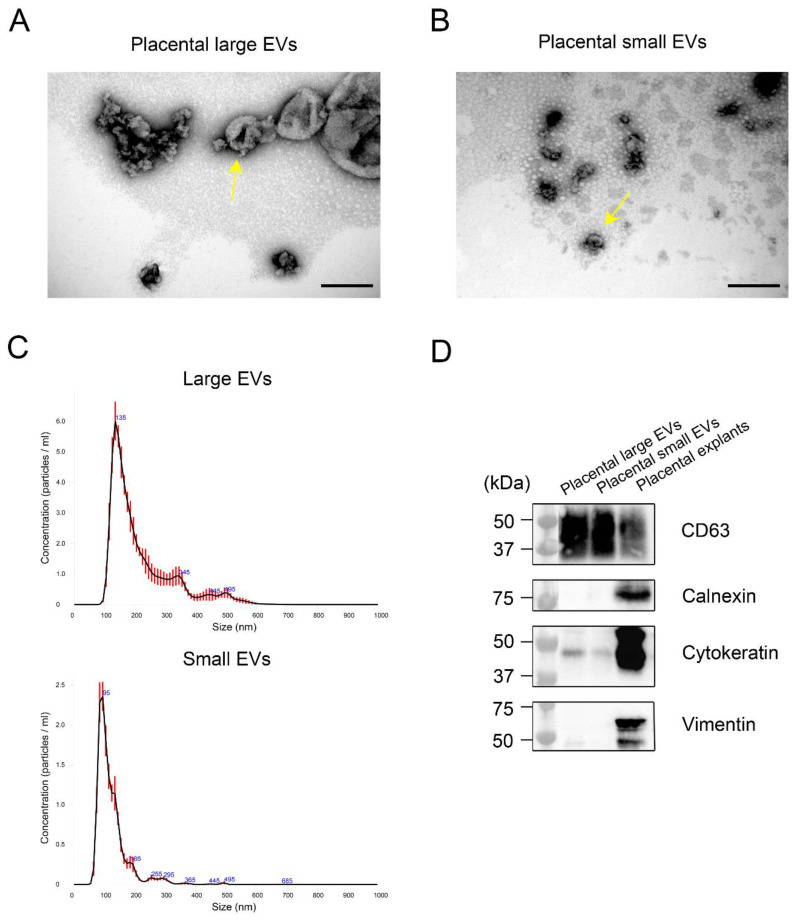
Characterization of the large- and small-extracellular vesicles (EVs) derived from the human term placentae. Representative electron micrograph of the characteristic morphology of the isolated large EVs with diameters of approximately 170 nm (yellow arrow) (**A**) and small EVs with diameters of approximately 60 nm (yellow arrow) (**B**), Scale bar = 200 nm. Nanoparticle tracking analysis of the size distribution in the large and small EVs, using the NanoSight NS300 system (**C**), error bars indicate ± 1 standard error of the mean ( *n* = 3 in each group). Representative western blots of specific markers present in the large and small EVs, derived from human placentae: CD63, Calnexin, Cytokeratin and Vimentin (**D**). A lysate of the placental explants served as a control.

Transmission electron microscopy (TEM) confirmed that placental large and small EVs had a typical spheroid or cup-shaped morphology (Figure 1A,B). NTA confirmed that the modal sizes of the large or small EVs were 146.66 ± 5.16 nm and 96.9 ± 3.74 nm, respectively (Figure 1C). Western blotting showed the presence of CD63 and cytokeratin and the absence of calnexin and vimentin in both the large and small placental EVs, suggesting the trophoblastic origin of these vesicles (Figure 1D).

### 2.2. The Interaction of the Placental EVs with Different Recipient Cells

**Figure 2:**
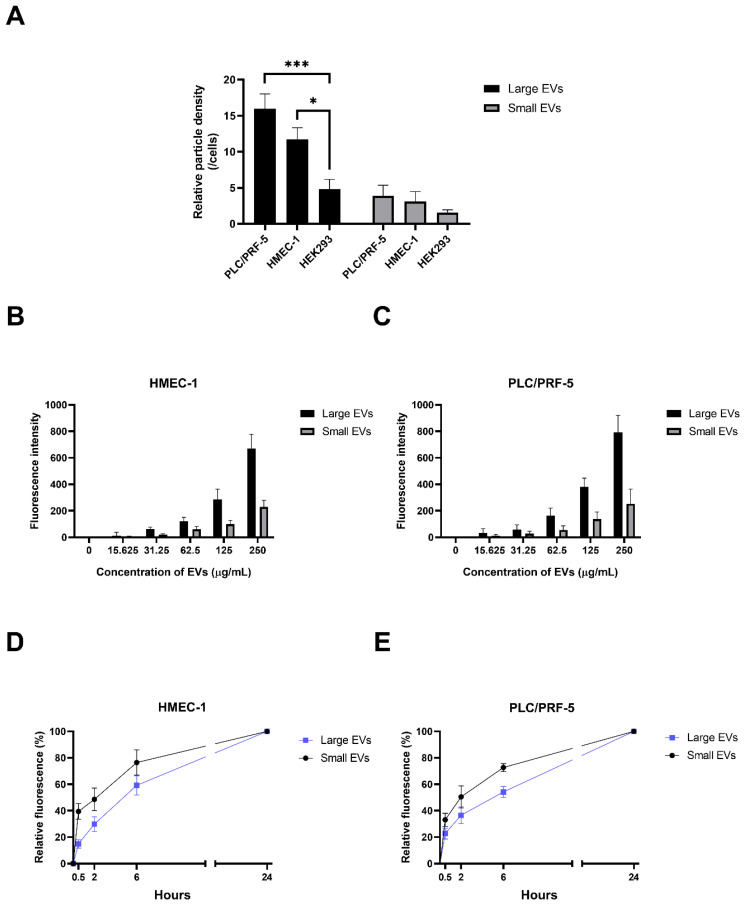
The interaction of the placental EVs with different recipient cells. (**A**) The interaction of the CellTracker^TM^ Red CMTPX-labelled placental large and small EVs with PLC/PRF-5, HMEC-1 or HEK293 recipient cells was examined by fluorscent microscopy and quantified using ImageJ 1.50i software (National Institutes of Health, Bethesda, MD, USA) (*n* = 3). Differences among the groups were tested with a RM one-way ANOVA with multiple comparisons (* *p* < 0.05, *** *p* < 0.001). Increasing doses of placental EVs, ranging from 0 to 250 µg/mL, were exposed to (**B**) HMEC-1 cells or (**C**) PLC/PRF-5 for 2 h (*n* = 4). Following the washing, the red fluorescence intensity was quantified using a BioTek Synergy 2 microplate reader (Agilent, Santa Clara, CA, USA). The time-dependence of the interaction of the CMTPX-labelled large and small placental EVs (250 µg/mL) with (**D**) HMEC-1 endothelial or (**E**) PLC/PRF-5 liver cells, was quantified by measuring the fluorescence intensity using a BioTek Synergy 2 microplate reader (Agilent, Santa Clara, CA, USA) and normalized to the reading at 24 h. Cells were exposed to the EVs for times ranging from 0.5–24 h (*n* = 4).

Our previous study showed that the liver and kidneys are the main organs to which the placental EVs are co-localised in vivo and that they have functional effects on the maternal vasculature [18,19]. To confirm the interaction of the placental EVs with the recipient cells, the red fluorescent-labelled placental EVs were co-cultured with HMEC-1 (endothelial cells), PLC/PRF-5 (liver cells) and HEK293 cells (kidney cells). As shown in Figure 2A, there was an interaction between the placental large or small EVs and all three types of recipient cells, after 2 h. We found that the interaction of the placental EVs was greatest with PLC/PRF-5, followed by the HMEC-1 cells with lesser interactions with the HEK293 cells. Given the limited interaction of the placental EVs and the HEK293 cells, we did not examine further the interactions of the EVs with these cells. To further investigate whether the interaction between the placental EVs and the recipient cells was energy-, dose- and/or time-dependent, the HMEC-1 and PLC/PRF-5 cells were treated with (1) increasing doses of placental EVs for 2 h, or (2) a constant dose of EVs (250 µg/mL) for increasing periods of time or (3) precooled cells were incubated with EVs at 4 °C for 2 h.

**Figure 3:**
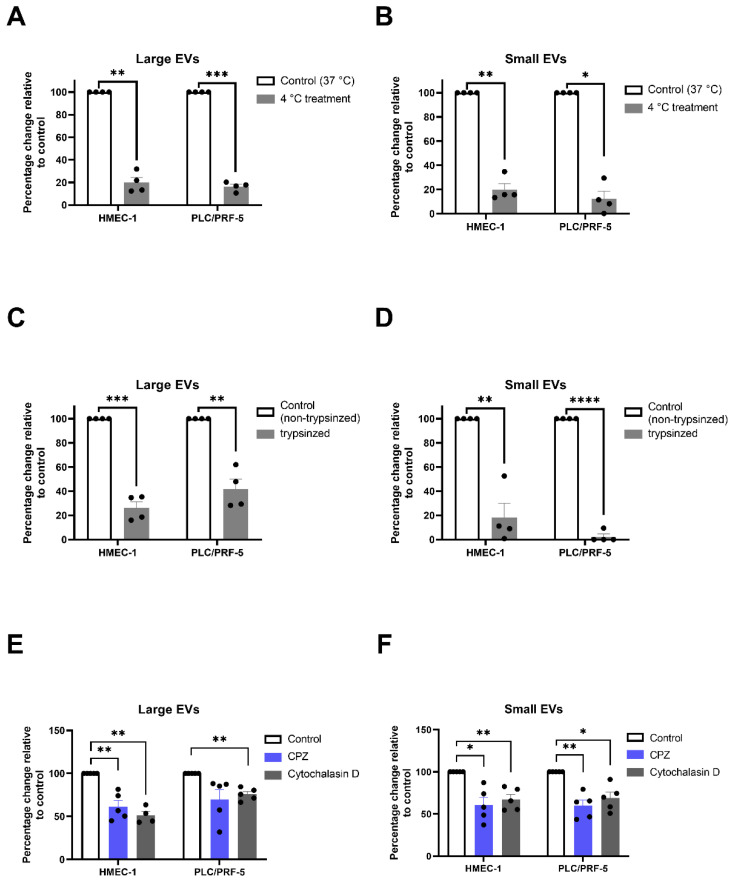
Inhibition of the interaction of the placental EVs with the recipient cells. HMEC-1 and PLC/PRF-5 were pre-cultured at 4 °C for 0.5 h. Fluorescently labelled placental (**A**) large or (**B**) small EVs were added, and the cultures continued for 2 h at 4 °C (*n* = 4). HMEC-1 and PLC/PRF-5 were treated with placental (**C**) large or (**D**) small EVs that had been treated with 0.25% (*w*/*v*) trypsin, for 2 h (*n* = 4). HMEC-1 and PLC/PRF-5 were pre-treated with cytochalasin D (10 µg/mL) or chlorpromazine hydrochloride (CPZ, 10 µg/mL) for 30 min, and the fluorescently labelled placental (**E**) large or (**F**) small EVs were added to the cultures in the presence or absence of the inhibitors for 2 h (*n* = 4–5). The interaction of the EVs was quantified using a BioTek Synergy 2 microplate reader (Agilent, CA, USA). Values were normalized to control EV group and shown as mean ± SEM. Differences between the two groups were tested with a paired *t*-test (* *p* < 0.05, ** *p* < 0.01, *** *p* < 0.001, **** *p* < 0.0001).

Incubation of both the HMEC-1 cells and the PLC/PRF-5 cells at 4 °C, significantly decreased the uptake of the fluorescently-labelled large and small EVs in the recipient cells, compared with the recipient cells co-cultured with EVs at 37 °C (Figure 3A,B), indicating an energy-dependent uptake mechanism.

The interaction of the placental EVs with HEMC-1 was increased as the dose of placental EVs increased, regardless of the size of the EVs (Figure 2B,C). In addition, the interactions of the placental EVs with HMEC-1 or PLC/PRF-5 cells, were time-dependent, but the interaction of the placental small EVs was faster than that of the large EVs (Figure 2D,E).

### 2.3. Both Phagocytosis and Endocytosis Were Involved in the Uptake of the Placental EVs by the Recipient Cells

We next investigated whether the proteins on the surface of the placental EVs were involved in the EV uptake. The placental large and small EVs were treated with trypsin for 30 min, then exposed to HMEC-1 or PLC/PRF-5 cells [20]. As shown in Figure 3, the uptake of both large and small EVs by the HMEC-1 cells (Figure 3C,D), and PLC/PRF-5 cells (Figure 3C,D) were significantly reduced following trypsinisation.

Phagocytosis and endocytosis have been suggested to be involved in the EV uptake [13]. To further investigate the potential mechanisms of the interaction between placental EVs and recipient cells, the HMEC-1 cells or PLC/PRF-5 cells were pre-treated with cytochalasin D (an inhibitor of phagocytosis) or chlorpromazine hydrochloride (CPZ, an inhibitor of the clathrin-mediated endocytosis) for 30 min, followed by adding red fluorescent-labelled placental large or small EVs into the cultures for 2 h. Adding CPZ or cytochalasin D into the cultures significantly reduced the uptake of both large (CPZ vs. Control, *p* < 0.05; cytochalasin D vs. Control, *p* < 0.01) and small EVs (CPZ vs. Control or cytochalasin D vs. Control, *p* < 0.05) by the HMEC-1 cells (Figure 3E,F). The cytochalasin treatment significantly reduced the uptake of both large and small EVs by the PLC/PRF-5 cells but CPZ only inhibited the uptake of the small, and not the large EVs by the PLC/PRF-5 cells (Figure 3E,F). While CPZ did cause a non-significant decrease in the uptake of the large EVs by the PLC/PRF-5 cells, this decrease was driven by an outlying data point.

**Figure 4:**
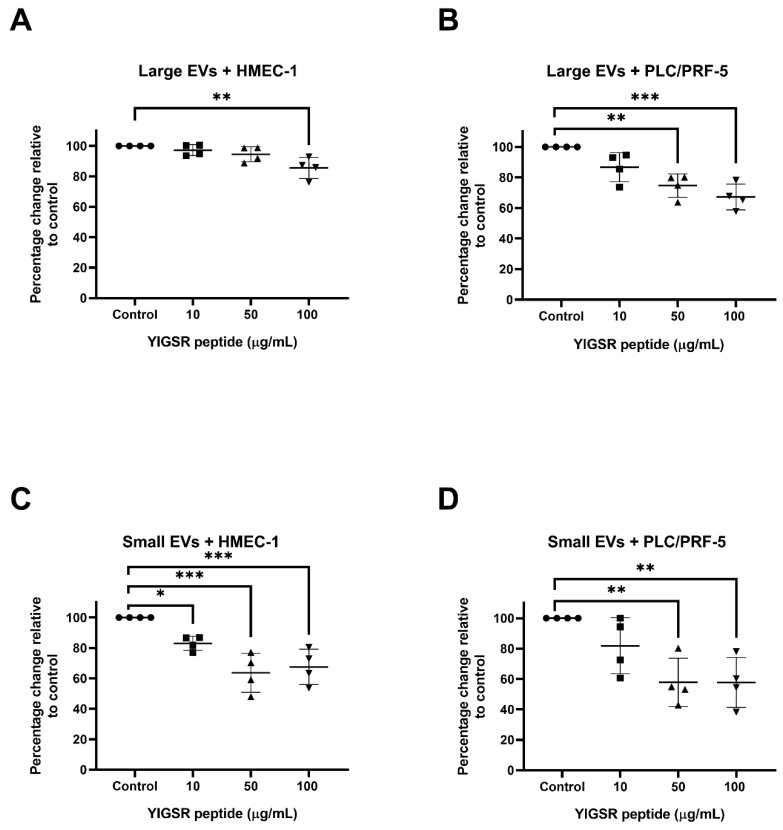
YIGSR peptide reduced the interaction of the placental EVs with the endothelial and liver cells. Fluorescently labelled placental large or small EVs were incubated with increasing concentrations of the YIGSR peptide (*n* = 4), and the interaction with the HMEC-1 or PLC/PRF-5 cells was quantified using a BioTek Synergy 2 microplate reader (Agilent, CA, USA). The interaction of the placental (**A**) large or (**C**) small EVs with the HEMC-1 cells was significantly reduced by the YIGSR peptide pre-treatment at the concentration of 100 µg/mL and 10 µg/mL, respectively. The interaction of the placental (**B**) large or (**D**) small EVs with the PLC/PRF-5 cells was significantly reduced by the YIGSR peptide pre-treatment at the concentration of 50 µg/mL compared to no YIGSR peptide pre-treatment. Values were normalized to the untreated HMEC-1 or PLC/PRF-5 cells. Data are shown as mean ± SEM. Differences among the groups were tested with a RM one-way ANOVA with multiple comparisons (* *p* < 0.05, ** *p* < 0.01, *** *p* < 0.001).

Our previous study reported that integrins were present in the placental large and small EVs [4] and other studies reported that integrins are involved in the uptake of EVs [16,21,22]. We used two well-known integrin-blocking peptides, RGD and YIGSR to investigate the interactions between the placental EVs and the recipient cells further [23,24]. RGD has been reported to block the ligand binding for multiple integrins while YIGSR blocks the β1-containing integrins, such as integrins α4β1 and α6β1. As shown in Figure 4, when the placental large EVs that had been treated with the YIGSR peptide, at 100 µg/mL for 2 h, were exposed to the HMEC-1 cells, the uptake of the EVs was significantly reduced (Figure 4A). In contrast, a two-fold lower dose of the YIGSR peptide significantly inhibited the uptake of the placental large EVs by the PLC/PRF-5 cells (Figure 4B). Treating the placental small EVs with the YIGSR peptide at 10 µg/mL, significantly inhibited the uptake of the EVs by the HMEC-1 cells (Figure 4C), but a five-fold higher concentration of the YIGSR peptide was required to significantly reduce the uptake of these EVs by the PLC/PRF-5 cells (Figure 4D). In contrast, the placental EVs that had been treated with the RGD peptide did not affect the EV uptake in either the HMEC-1 or PLC/PRF-5 cells (Supplementary Figure S1A–D).

### 2.4. Blocking the Integrin-Mediated Uptake of the Preeclamptic Placental EVs Reversed Their Adverse Effects on the Endothelial Cells

**Figure 5:**
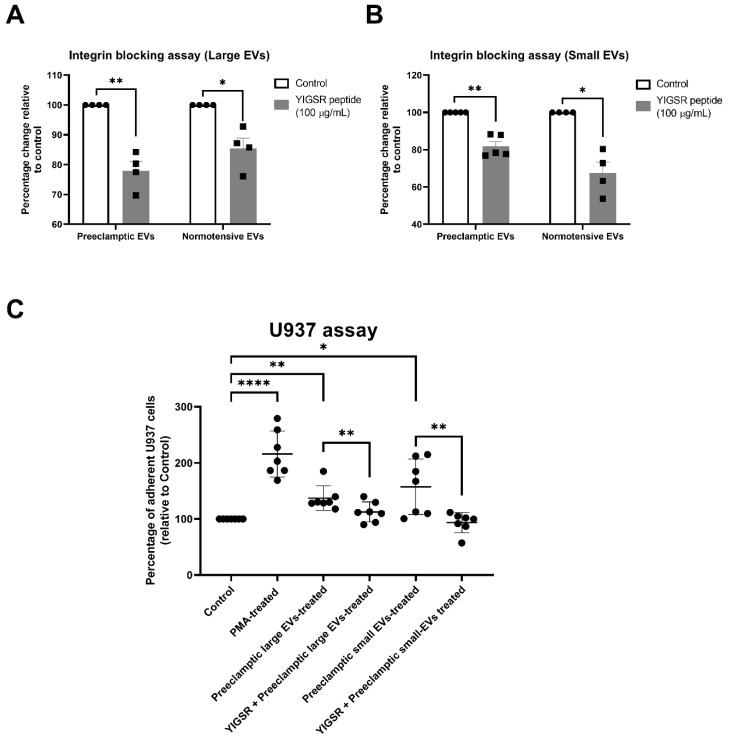
Blocking the integrin-mediated uptake of the preeclamptic placental EVs reversed their adverse effects on the endothelial cells. Fluorescently labelled placental (**A**) large EVs and (**B**) small EVs from preeclamptic placentae (*n* = 5) or normal placentae (*n* = 4), were pre-treated with the YIGSR integrin-blocking peptide (100 µg/mL). The interaction of the EVs with the HMEC-1 cells was quantified using a BioTek Synergy 2 microplate reader (Agilent, CA, USA). The YIGSR peptide significantly reduced the interaction of both the large and small EVs, from both the normotensive (*p* = 0.025 and *p* = 0.024, respectively) and preeclamptic placentae (*p* = 0.01 and *p* = 0.006, respectively). (**C**) Endothelial cell activation, in response to the large or small preeclamptic EVs, was measured by quantifying the adhesion of the fluorescently labelled U937 monocytes to the endothelial cells (*n* = 7). Endothelial cells treated with PMA were used as a positive control for the activation. The increased activation of the HMEC-1 cells induced by both the large and small preeclamptic placental EVs, was inhibited by the preincubation with the YIGSR peptide. Values were normalized to the untreated HMEC-1 cells, and data are shown as mean ± SEM. Differences between the two groups were tested with a paired *t*-test (* *p* < 0.05, ** *p* < 0.01, **** *p* < 0.0001).

It has previously been shown that EVs derived from preeclamptic placentae, activated the endothelial cells, as indicated by the increased adhesion of the monocytes to the endothelial cells [25,26]. To examine whether the integrins are important in the uptake of the placental EVs from preeclamptic placentae, we investigated the uptake of EVs derived from preeclamptic placentae into endothelial cells, following a pre-treatment with the YIGSR peptides. Blocking the integrin-mediated uptake resulted in a 20% (large EVs) and 15% (small EVs) reduction in the uptake of preeclamptic EVs by the endothelial cells (Figure 5A, *p* < 0.05), while the uptake of the normotensive placental large and small EVs, were reduced by 15% and 32%, respectively.

We then investigated whether the pre-treatment of the preeclamptic EVs with the YIGSR peptide could reverse the activation of the endothelial cells, induced by the preeclamptic placental EVs. The uptake of the preeclamptic large EVs induced a small increase (1.37-fold, *p* < 0.001) in the monocyte adhesion to the HMEC-1 cells that was reversed by the YIGSR peptide (Figure 5B, *p* < 0.01). Whereas, small preeclamptic EVs induced a larger increase (1.57-fold, *p* < 0.05) in the monocyte adhesion, which was also reversed by pre-treating the EVs with the YIGSR peptide (Figure 5B, *p* < 0.01). As a positive control, we activated the endothelial cells with PMA.

## 3. Discussion

In recent years, the manner of the uptake of EVs by the recipient cells, has been investigated in vitro and in vivo, as EVs can be internalised by the recipient cells with the subsequent release of cargos that EVs carry, facilitating the impacts of the EVs on the functions of the recipient cells [15]. During pregnancy, a large number of placental EVs are extruded into the maternal circulation [1,27,28] and phagocytosis of these EVs by the vascular endothelial cells contributes to the maternal vascular adaptation [18,29]. Our previous study reported that the liver and kidneys are the main organs to which the placental EVs are localised, in vivo, regardless of the subtype of placental EVs [18]. In this study, we confirmed that the maternal endothelial cells, liver and kidney cells are capable of taking up placental EVs. The greatest uptake of the placental EVs was seen in the liver cells, followed by the endothelial cells. Kidney cells had a lesser uptake of the placental EVs, regardless of the size of the EVs. Although we do not know the exact reasons for the difference in the uptake rate between the recipient cells, a recent systematic analysis reported that, regardless of the size of the EVs, the biodistribution of the EVs was largely seen in the liver and with fewer in the kidneys [30]. In our current study, we found that the uptake rate of large EVs was greater than the uptake rate of small EVs, by the cells we tested. The efficiency of the EV uptake was cell-specific and EV size-dependent. However, here we cannot make a valid comparison of this difference, as the large EVs may contain a more fluorescent label than that in the small EVs, and the uptake rate was measured by the intensity of fluorescence in the recipient cells. Further study is required to investigate this observation.

In this study, we also found that the uptake of small EVs was faster than the uptake of large EVs, by both endothelial cells and liver cells. This observation supports other studies showing that smaller EVs (<100 nm) can be taken up by recipient cells more rapidly [31]. This may be due to the different proteins present on the surface of the EVs between the large and small EVs [4]. Regardless of the EV size, the majority of the uptake by the endothelial and liver cells, occurred within the first 6 h of exposure to the EVs (Figure 2D,E).

It was previously shown that the internalization and processing of EVs into cells is energy-dependent [9,32,33]. In this study, we confirmed this energy-dependence of the EV uptake process, showing the decreased interaction of the placental EVs and the recipient cells at 4 °C, regardless of whether the EVs were large or small. This inhibition of the EV-cell interaction by low temperatures, suggested an active process of the placental EV uptake, rather than a passive process.

A variety of mechanisms that may underly the uptake of EVs have been proposed including, clathrin-mediated endocytosis (CME), phagocytosis, micropinocytosis and plasma or endosomal membrane fusion [13]. In our current study, we found that the uptake of placental EVs by endothelial or liver cells was reduced by 35–40%, in the presence of chlorpromazine (CPZ), an inhibitor of the clathrin-mediated endocytosis. A similar level of inhibition by cytochalasin D, an inhibitor of phagocytosis, suggesting both the clathrin-mediated endocytosis and phagocytosis are involved in the placental EV uptake. This is consistent with our previous report that both processes are involved in the EV uptake into the endothelial cells [11]. Our preparations were enriched for the large or small EVs but they are not homogenous populations and it has been suggested that a heterogeneous population of EVs could be internalized by a cell, via more than one route [13]. Although a recent study reported that phagocytosis was not involved in the placental EV uptake by the endothelial cells, using cytochalasin D [9], this could be because the methods of measuring the EV uptake were different between the two studies. In the Cronqvist et al. study, the EVs were labelled with PKH, which is a lipid membrane dye. However, using membrane stains have the potential to affect the behaviour of EVs and also to leach from the EV membrane into other cellular structures, consequently leading to different patterns of cellular staining following the uptake [34]. While, in our current study, we used a stain that is sequestered in the cytoplasm and is less likely to affect the interactions between the EVs and cells.

In addition to the endocytosis or phagocytosis mechanism of the EV uptake, the interaction between the specific proteins on the surface of the EVs and proteins on the surface of the recipient cells has also been investigated [15] and several EV membrane surface proteins have been shown to play major roles in the cellular targeting and uptake [13]. In this study, when the surface proteins on the placental EVs were removed by the treatment with trypsin, the uptake of the placental EVs was abolished, suggesting proteins on the surface of the placental EVs are also involved in the uptake of the placental EVs.

Integrins have been implicated in the interaction between EVs and recipient cells mediating the attachment and facilitating the internalisation [35,36,37]. Placental EVs carry a large range of integrins, including integrins α1, αIIb, α5, α6, αM, β1, β2, β3 and β5 [4]. Integrin-blocking peptides have been widely used to block the integrin-ligand interactions [33,38,39,40,41]. In our current study, treating the placental EVs with the YIGSR, but not the RGD peptide, significantly inhibited the uptake of the placental EVs by both the endothelial and liver cells, regardless of the size of the EVs. Our data suggests that the integrin mediated pathways are involved in, at least in part, the interaction of the placental EVs with target cells. Both the RGD and YIGSR peptides act as inhibitors of the integrin-ligand interactions [42]. While the RGD peptide, binds to integrins αvβ3, αvβ5, αvβ6, αvβ1, αvβ8, α5β1, αIIbβ3 and α8β1 [43], the YIGSR peptide derived from laminin is known to interact with integrins containing the β1 chain. It has been reported that pre-treating tumour-derived EVs with an RGD peptide, resulted in a significant reduction of the EV distribution to the liver [16,35]. The most obvious explanation for the difference between the results of Nguyen et al. and our study, is the different origin of the EVs studied. Nguyen et al. have shown that pre-treating murine placental EVs with an RGD peptide, reduced the distribution of these vesicles to the liver, in vivo, but they showed that the murine EVs were co-localised with endothelial cells and Kupffer cells [16]. Whereas, in our study we have examined the interaction of the human placental EVs with cells that are derived from the liver epithelium and a combination of the species differences between the studies, cellular and in vivo, versus in vitro methodologies, may explain the differences in our results. We found that the YIGSR peptide has a stronger inhibitory effect on the interaction with liver cells, compared to the endothelial cells (Figure 4), suggesting that the pathway of the placental EV interactions with the recipient cells is dependent on the integrins present on the EVs and/or the ligands expressed by the target cells, reinforcing the concept that EVs are preferentially targeted to specific organs/cells.

Several studies have suggested that the protein content, including the surface proteins on EVs, derived from preeclamptic placentae are altered [6,25,44,45,46]. These changes affect the EV uptake and the subsequent changes in the function of the target cells. We have previously shown that the preeclamptic but not the normotensive placental EVs, induce the activation of endothelial cells, which can be quantified by measuring the monocyte adhesion to the endothelial cells [25,26]. Here, we have confirmed that the preeclamptic EVs were bioactive and that treating the endothelial cells with preeclamptic placental EVs, caused an endothelial cell activation. We found that while pre-treating preeclamptic EVs with the YIGSR peptide did reduce the interaction of the EVs with the HMEC-1 endothelial cells, the blocking effect of YIGSR on the interaction of the preeclamptic small EVs with the HMEC-1 cells, was significantly less than the blocking effect seen with the normal placental EVs, suggesting there may be fewer relevant integrin molecules on the preeclamptic EVs than the normal EVs. Despite the reduced effect of YIGSR on blocking the EV-HMEC-1 interactions, we nevertheless found that blocking the interaction between the preeclamptic EVs and the HMEC-1 cells with YIGSR, reversed the activation of the endothelial cells induced by the EVs derived from preeclamptic placentae. This confirms that the integrins are important in the interaction of preeclamptic EVs with endothelial cells, and that by blocking this interaction, we could reduce the harmful biologic effect of preeclamptic EVs.

In conclusion, our data demonstrate that multiple pathways, including endocytosis, phagocytosis and protein interactions between vesicular integrins and cellular ligands are involved in the mechanisms of placental EV targeting and uptake, regardless of the size of EVs. The blocking of integrin-mediated interactions with maternal endothelial cells could reverse the endothelial cell dysfunction induced by the EVs derived from preeclamptic placentae.

## 4. Materials and Methods

### 4.1. Ethical Approval

The collection of human placentae for this study was approved by the Auckland Regional Health and Disabilities Ethics Committee (NTX/12/06/057/AM11). Normotensive placentae (*n* = 35) were collected from Auckland City Hospital (Auckland, New Zealand (NZ)) within five hours of birth. Preeclamptic placentae (*n* = 7) were collected from women who were diagnosed with preeclampsia from Auckland City Hospital (Auckland, New Zealand), followed by the international guidelines [47]. All placentae were collected with informed written consent.

### 4.2. Cell Culture

Human microvascular endothelial cells (HMEC-1) (American Type Culture Collection (ATCC), CRL3243, Manassas, VA, USA) were cultured in MCDB-131 medium (Life Technologies, Inchinnan, UK) complemented with 10% FBS (Thermo Fisher, San Jose, CA, USA), 1% L-Glutamine and 1% Penicillin/Streptomycin, at 37 °C 5% CO_2_. Human liver PLC/PRF-5 cells (ATCC, CRL8024, Manassas, VA, USA) were grown in RPMI-1640 medium (Life Technologies, Inchinnan, UK), supplemented with 10% FBS and 1% Penicillin/Streptomycin. Human Kidney HEK293 cells, were grown in DMEM medium (Thermo Fisher, San Jose, CA, USA), supplemented with 10% FBS and 1% Penicillin/Streptomycin. Human monocytes U937 cells were (ATCC, CRL1593.2, Manassas, VA, USA) cultured in RPMI-1640 (Life Technologies, Inchinnan, UK) medium complemented with 10% FBS and 1% Penicillin/Streptomycin.

### 4.3. Isolation and Labelling of the Placental Extracellular Vesicles

Placental large and small EVs were collected from human term placentae, using a well-established explant culture model [48]. Briefly, the placental tissues were dissected into explants of 400 mg wet weight and washed thoroughly to remove the contaminating blood. Then, the explants were cultured in 12-well culture plates with Netwell inserts (400 µm, Corning, Glendale, AZ, USA) in Advanced DMEM/F12 medium (In Vitro Technologies, Auckland, NZ), complemented with 2% FBS and 1% P/S overnight. The cultured medium was collected and centrifuged sequentially at 4 °C as follows: 2000× *g* for five minutes to remove the macro-vesicles/syncytial nuclear aggregates (SNAs) and cellular debris, 20,000× *g* for 60 min to collect large EVs, and 100,000× *g* for 60 min to collect small EVs (Sorvall wX+ ultracentrifuge (Thermo Fisher, San Jose, CA, USA)). The EV pellets were resuspended in PBS and were stored at 4 °C for future use.

In some experiments, the human placental explants were cultured in the presence of CellTracker^TM^ Red CMTPX (1 µg/mL, Thermo Fisher, Eugene, OR, USA) to generate fluorescently labelled placental large and small EVs.

### 4.4. Nanoparticle Tracking Analysis (NTA)

The concentration of placental large and small EVs and the size distribution were measured using a NanoSight NS300 (Malvern Panalytical, Almelo, The Netherlands) with a camera level set at 13 and a detection threshold at 6. Samples were measured in triplicate using the NanoSight NS300 system. Five videos (30 s duration each) of the Brownian motion of the nanoparticles were recorded and analysed. The samples were measured with a manual shutter and gain adjustments three times.

### 4.5. Transmission Electron Microscopy (TEM)

The placental large and small EVs were viewed on the Tecnai™ G2 Spirit Twin TEM (FEI, Tokyo, Japan), as described previously [49]. Briefly, samples were adsorbed to a Formvar-coated 150 copper mesh grid (Electron Microscopy Sciences, Hatfield, PA, USA) and negatively-stained with 2% uranyl acetate, followed by a washing step using distilled water. Images were taken at 97,000× magnification by the Morada Camera (Soft Imaging Systems, Münster, Germany) and processed using the iTEM software (Soft Imaging Systems, Münster, Germany).

### 4.6. Western Blotting

The placental EVs were characterised by measuring the small EV markers CD63, pan cytokeratin (a marker of trophoblasts), and the markers of contamination vimentin and calnexin. EVs were resuspended in fresh ice-cold radioimmunoprecipitation (RIPA) buffer (50 mM Tris, 150 mM NaCl, 1% sodium deoxycholate, 0.1% sodium dodecyl sulphate (SDS), 1% Nonidet P40 substitute, 1 mM phenylmethylsulfonyl, fluoride protease inhibitor (Roche Diagnostics Deutschland GmbH, Mannheim, Germany), pH 7.4) and were incubated for 30 min on ice, and the proteins were collected by centrifugation after removing the pelleted debris. In addition, the placental explants were homogenised using a Q700 Sonicator (QSonica, Newtown, CT, USA) in ice-cold RIPA buffer for 30 s before incubation on ice. Then, the samples were centrifuged at 13,000× *g* for 10 min, and the supernatants were collected to be used as controls. The concentration of the protein levels in the placental large and small EVs, and the placental extracts were measured by Pierce^TM^ BCA assay (catalog number: 23,225, Thermo Fisher, San Jose, CA, USA), following the manufacturer’s instructions. The protein lysates were mixed with 5 × reducing or non-reducing Laemmli buffer and boiled at 97 °C for 5 min. Then, 20 µg of each sample was loaded and resolved on 4–15% sodium dodecyl sulphate polyacrylamide gel electrophoresis (SDS-PAGE) gels. The resolved proteins were transferred onto PVDF membranes using a Criterion Blotter system (Bio-Rad Laboratories, Hercules, CA, USA). The membranes were blocked in blocking buffer (5% non-fat dry milk in PBST (0.1% Tween20)) and gently rocked for one hour at room temperature. Then, the blots were incubated with primary antibodies (Rabbit anti-human CD63 (Abcam, Cambridge, UK, 1:1000), Rabbit anti-human Calnexin (Abcam, Cambridge, UK, 1:1000), Mouse anti-human cytokeratin (Agilent DAKO, Santa Clara, CA, USA,, 1:1000), Mouse anti-human vimentin (Agilent DAKO, Santa Clara, CA, USA, 1:1000)), in a blocking buffer overnight at 4 °C, washed and incubated with horseradish peroxidase (HRP)-conjugated secondary antibody (Streptavidin conjugated goat anti-mouse or anti-rabbit IgG antibody, Jackson ImmunoResearch, West Grove, PA, USA, 1:5000) in PBST. The blots were developed using the Amersham ECL^TM^ Prime detection reagent (Cytiva, Buckinghamshire, UK) and visualised on an ImageQuant LAS-3000 (Fujifilm, Tokyo, Japan). The image analysis and quantification were performed using Image Lab Version 6.1.0 Software (Bio-Rad lifeLaboratories, Hercules, CA, USA) and Adobe Photoshop 21.2.3 software (Adobe Inc., San Jose, CA, USA).

### 4.7. Detection and Quantification of the Uptake of Placental EVs

Three recipient cell types, HMEC-1 (endothelial), PLC/PRF-5 (liver) and HEK293 (kidney) were seeded at a density of 10,000 cells per well in a 96-well plate and were grown overnight. The recipient cells were labelled with CellTracker^TM^ Green CMFDA (0.5 µg/mL, Thermo Fisher, Eugene, OR, USA) for 2 h and the fluorescent labelled placental EVs (red) were then added to the recipient cells in the serum-free culture medium at a final concentration of 250 µg/mL of protein at 37 °C. The cells were extensively washed with PBS at each end point to remove the unbound EVs and fixed with 4% paraformaldehyde for 15 min. The cell nuclei were counterstained with Hoechst 33342 (Sigma Aldrich, Saint Louis, MO, USA) when required. Five images from each culture were taken randomly using the same camera and microscope settings (Nikon Eclipse E400 Fluorescence Microscope (Nikon, Tokyo, Japan), with a Nikon DS-Ril digital camera (Nikon, Tokyo, Japan)). Vesicles appeared as red punctate structures attached or inside the cells. The uptake rate of the placental EVs by the recipient cells was then quantified using ImageJ 1.50i software (National Institutes of Health, Bethesda, MD, USA), as previously described [50].

In some experiments, the different doses of placental EVs, ranging from 0 to 250 µg/mL were exposed to HMEC-1 cells and PLC/PRF-5 for 2 h. Following a washing with PBS for three times to remove the unbounded EVs, the red fluorescence intensity was quantified using a BioTek Synergy 2 microplate reader (Agilent, CA, USA), considered as an uptake rate of the placental EVs. In some experiments, the labelled placental EVs (250 µg/mL) were exposed to HMEC-1 cells and PLC/PRF-5 and the cultures were continued for times ranging from 0.5–24 h. At each time point, after washing with PBS for three times, the fluorescence intensity was quantified using a BioTek Synergy 2 microplate reader (Agilent, CA, USA).

In some experiments, HMEC-1 and PLC/PRF-5 were pre-treated with cytochalasin D (10 µg/mL, Sigma Aldrich, Saint Louis, MO, USA) or chlorpromazine hydrochloride (CPZ, 10 µg/mL, Sigma Aldrich, Saint Louis, MO, USA) for 30 min or were pre-cultured at 4 °C for 30 min, and the fluorescently labelled placental EVs were added in the cultures in the presence or absence of the inhibitors for 2 h. In some experiments, placental EVs were incubated with 0.25% (*w*/*v*) trypsin solution (Life Technologies, Inchinnan, UK) at 37 °C for 30 min, as previously described [51], or RGD (Arg-Gly-Asp) or YIGSR (Tyr-Ile-Gly-Ser-Arg) peptide (Sigma Aldrich, Saint Louis, MO, USA), at concentrations ranging from 0–100 µg/mL, at 37 °C for 2 h. Then, the samples were centrifuged again at 20,000× *g* for the large EV and 100,000× *g* for the small EV collections. HMEC-1 and PLC/PRF-5 were treated with placental EVs that had been treated with trypsin or RGD or YIGSR peptide, respectively, for 2 h. The fluorescence intensity was then quantified using a BioTek Synergy 2 microplate reader (Agilent, CA, USA), considered as an uptake rate of the placental EVs.

### 4.8. Monocyte Adhesion Assay

HMEC-1 endothelial cell activation was measured using a monocyte adhesion, as described previously [25,52] The pre-treatment EVs derived from preeclamptic placentae with YIGSR peptide (100 µg/mL) for 2 h. Following a washing out of the peptide, the EVs were exposed to confluent HMEC-1 cells in quadruplicate, for 24 h at 37 °C. Cells were then washed, and CellTracker^TM^ Green CMFDA-labelled U937 monocytes were added (1 × 10^4^ monocytes/well of a 96-well plate). The monocytes and HMEC-1 cells were co-cultured for 6 h at 37 °C. Then, the unbound monocytes were thoroughly washed off with PBS, and the adherent monocytes were measured using a BioTek Synergy 2 microplate reader (Agilent, CA, USA) at Excitation/Emission: 485/20,528/20 nm. The fluorescence intensity was expressed as a percentage of the adherent U937 cells to HMEC-1, compared to the untreated cells. As a positive control, we activated the endothelial cells with Phorbol 12-myristate 13-acetate (PMA, 10 ng/mL; Sigma Aldrich, Saint Louis, MO, USA).

### 4.9. Statistical Analysis

A statistical analysis was performed using GraphPad 8.2.1 (441) software (GraphPad Software Inc., San Diego, CA, USA). The data was normalized to the control group. The differences between the two groups were tested with the paired *t*-test, while differences among three or more groups were analysed using repeated measures one-way analysis of variance (RM one-way ANOVA) with multiple comparisons. The statistical significance was determined by *p* value < 0.05.

## Data Availability

The data used to support the findings of the present study are available from the corresponding author upon request.

## Supplementary Materials

The following supporting information can be downloaded at: https://www.mdpi.com/article/10.3390/ijms232113115/s1.
