# Supplementary material for: The Blocking of Integrin-Mediated Interactions with Maternal Endothelial Cells Reversed the Endothelial Cell Dysfunction Induced by EVs, Derived from Preeclamptic Placentae
Source: Int J Mol Sci. 2022 Oct 28;23(21):13115. doi: 10.3390/ijms232113115 (PMC9657319; doi:10.3390/ijms232113115)
Supplement: Supplementary file 1 [file ijms-23-13115-s001.zip › ijms-1991955-supplementary.pdf]

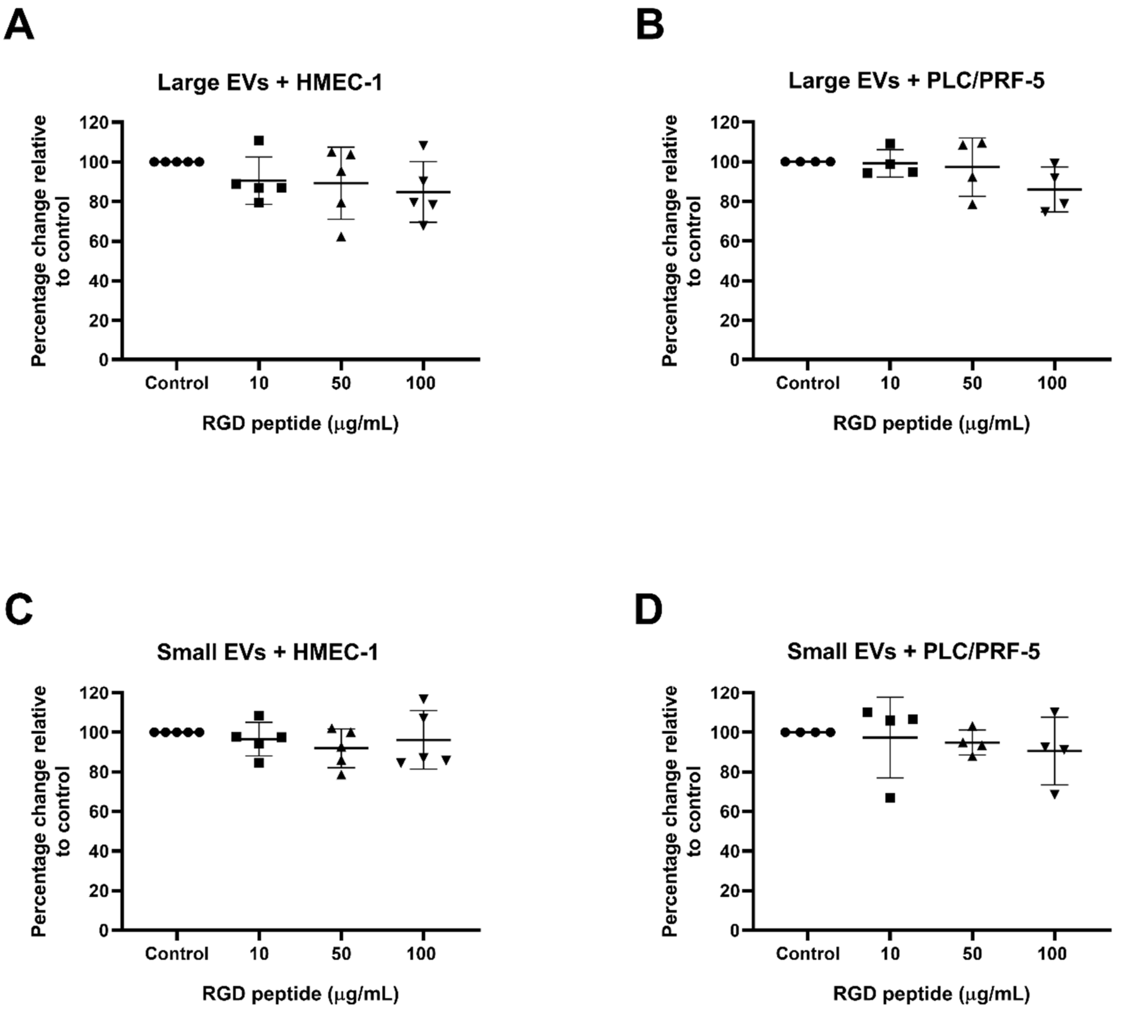

Supplementary Figure S1. RGD peptide did not reduce the interaction of placental EVs with endothelial or liver cells. Fluorescently labelled placental large or small EVs were incubated with increasing concentrations of the RGD peptide (n = 4-5) and the interaction with HMEC-1 or PLC/PRF/5 cells was quantified using a Synergy 2 microplate reader (BioTek, NZ). Values were normalized to untreated HMEC-1 or PLC/PRF/5 cells. Data are shown as mean ± SEM and there were no significant differences. Differences among the groups were tested with a repeated measure one-way ANOVA corrected for multiple comparisons.
